# Acute Monoblastic Leukemia (AML-M5) Presenting As Tonsillar Hypertrophy: An Unusual ENT Presentation

**DOI:** 10.7759/cureus.106709

**Published:** 2026-04-09

**Authors:** Nour El Houda Fakhri, Karima Ryouni, El Alaoui Mounia, Noufissa Benajiba

**Affiliations:** 1 Pediatrics, Hôpital Mère-Enfant Abderrahim Harouchi, Casablanca, MAR; 2 Hematology and Oncology, Hôpital Mère-Enfant Abderrahim Harouchi, Casablanca, MAR; 3 Pediatric Hematology, Centre Hospitalier Universitaire Mohammed VI de Oujda, Oujda, MAR

**Keywords:** acute myelomonocytic leukemia, aml-m5, obstructive sleep apnea syndrome, tonsillar hypertrophy, trisomy of 21

## Abstract

Acute monoblastic leukemia (AML-M5) is a rare subtype of acute leukemia in children and may present with extramedullary involvement. We report the case of a nine-year-old boy with trisomy 21 who was referred to the otorhinolaryngology department for severe obstructive sleep apnea syndrome (OSAS). Clinical examination revealed marked bilateral tonsillar hypertrophy without associated signs of infection. Preoperative laboratory investigations performed prior to planned adenotonsillectomy demonstrated bicytopenia and circulating blasts on the peripheral blood smear. Bone marrow aspiration confirmed the diagnosis of AML, subtype M5. The patient was managed in the pediatric hematology and oncology department at Harouchi University Hospital in Casablanca. Following initiation of chemotherapy, a spontaneous regression of the tonsillar hypertrophy was observed, confirming its leukemic origin. This case highlights the importance of considering an underlying hematologic malignancy in cases of atypical tonsillar hypertrophy, particularly in children with known risk factors.

## Introduction

Acute monoblastic leukemia (AML-M5) is a rare subtype of acute myeloid leukemia characterized by a predominant proliferation of monoblasts and/or promonocytes in the bone marrow, with a known tendency for extramedullary infiltration. In pediatric patients, the initial clinical presentation is often nonspecific and commonly includes manifestations related to bone marrow failure, such as anemia, thrombocytopenia, recurrent infections, fever, or bone pain. These features may delay diagnosis, particularly in the absence of overt hematologic abnormalities [1,2].

In some cases, acute monocytic leukemia may present with isolated extramedullary involvement, affecting soft tissues and mucosal sites [3,4]. Orofacial involvement is among the recognized presentations, most frequently involving the gingiva. However, isolated tonsillar infiltration as the initial manifestation of AML-M5 remains exceptionally uncommon. When tonsillar hypertrophy is prominent, it is typically attributed to infectious or reactive lymphoid causes, especially in children, and is rarely suspected to be of malignant origin.

The occurrence of massive tonsillar hypertrophy leading to obstructive sleep apnea syndrome (OSAS) represents a particularly misleading clinical scenario. OSAS is a frequent condition in the pediatric population and is most often associated with benign adenotonsillar hypertrophy. Consequently, the presence of severe obstructive symptoms may divert attention away from systemic causes and delay appropriate diagnostic evaluation.

We report a rare case of a child with bilateral tonsillar hypertrophy causing severe obstructive sleep apnea, in whom further investigation unexpectedly led to the diagnosis of acute monocytic leukemia. This observation emphasizes the importance of considering hematologic malignancies in the differential diagnosis of atypical, rapidly progressive, or unexplained tonsillar hypertrophy, particularly in children with predisposing conditions or without clear signs of infection or inflammation.

## Case presentation

A nine-year-old boy with trisomy 21 and no known history of hematologic disease was referred to the otorhinolaryngology department for significant nocturnal respiratory discomfort, associated with loud snoring, witnessed apneic episodes during sleep, and excessive daytime sleepiness, evolving over a three-week period in an afebrile context. Based on the clinical findings, severe OSAS was suspected.

Otorhinolaryngologic examination revealed grade IV bilateral tonsillar hypertrophy, nearly completely obstructing the oropharynx, without marked inflammatory signs, associated pain, or gingival hypertrophy. No acute infectious focus was identified. In view of the severity of the obstructive symptoms and the high number of apneic episodes, the patient was scheduled for urgent tonsillectomy.

Preoperative laboratory evaluation revealed bicytopenia, with anemia (hemoglobin level of 10 g/dL) and thrombocytopenia (platelet count of 86,000/L), as well as leukocytosis with monocytic predominance and the presence of 60% circulating blasts. Peripheral blood smear examination confirmed the presence of blasts (Table 1).

**Table 1:** Initial preoperative blood workup MCV: mean corpuscular volume; MCHC: mean corpuscular hemoglobin concentration; WBC: white blood cells

Parameter	Result	Unit of measure	Reference range
Hemoglobin	10	g/dL	12.0-15.0
MCV	81	f/L	77-95
MCHC	29	g/dL	32-36
WBC	29000	/µL	4.5-13.5 ×10^9^/L
Neutrophils	3000	/µL	1.5-8.0 ×10^9^/L
Lymphocytes	3500	/µL	1.2-5.2 ×10^9^/L
Platelets	86000	/L	150-450 ×10^9^/L
Blasts	59	%	0

**Figure 1:**
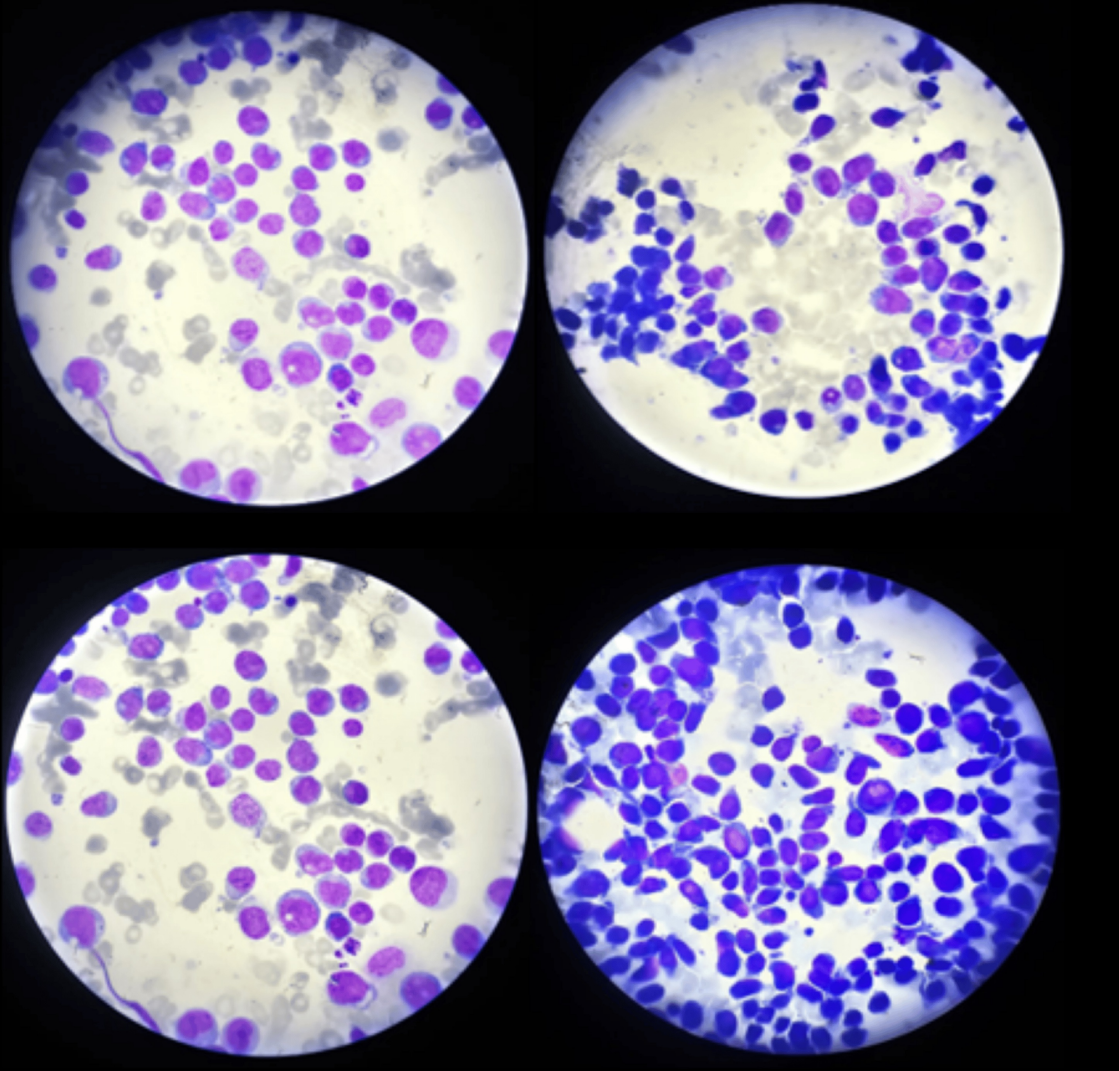
Bone marrow aspiration of the patient demonstrating acute monoblastic leukemia, subtype M5, with predominant monoblastic infiltration Bone marrow aspirate stained with a Romanowsky-type stain: Wright-Giemsa stain (magnification: 1000× total).

Bone marrow aspiration confirmed the diagnosis of AML, subtype M5 according to the French-American-British (FAB) classification, with predominant monoblastic infiltration (Figure 1).

Chemotherapy was initiated according to the established treatment protocol. The initial course was notable for a progressive regression of the tonsillar hypertrophy under systemic therapy, followed by complete remission as confirmed on multiple follow-up bone marrow evaluations. At present, the patient remains in complete remission after completion of chemotherapy, with ongoing monthly surveillance.

## Discussion

Gingival hypertrophy is a well‑recognized extramedullary manifestation of acute leukemias, particularly the myelomonocytic and monocytic subtypes (FAB M4/M5), due to the high affinity of leukemic cells for vascularized connective tissues where endothelial adhesion molecules facilitate leukocyte infiltration and proliferation [1,2]. Oral involvement may sometimes precede hematologic diagnosis, prompting dental or medical evaluation [2]. Although gingival infiltration is well documented, extramedullary invasion involving other lymphoid tissues, such as the tonsils, is exceptionally rare and may be mistaken for benign otolaryngologic pathology like reactive lymphoid hyperplasia or infection [3]. In the case presented, the child’s severe OSAS, commonly linked with reactive lymphoid hypertrophy in pediatric populations, initially reduced suspicion of malignancy [3].

The pathogenesis of leukemic tissue infiltration involves both blast proliferation in extramedullary sites and interaction with local microvascular and stromal elements; gingival tissues appear especially susceptible because of their abundant vascular network and expression of adhesion molecules that facilitate leukocyte extravasation [2]. Similar mechanisms likely underlie rare tonsillar involvement, although the tonsillar lymphoid architecture may make this less common. The rapid growth of leukemic infiltrates in lymphoid tissues underscores the importance of considering hematologic malignancy in cases of atypical lymphoid hypertrophy that progress quickly without a clear infectious etiology.

Diagnosis of extramedullary leukemic involvement hinges on a high index of suspicion and multidisciplinary evaluation. Complete blood count and peripheral smear may reveal leukocytosis with circulating blasts, prompting urgent bone marrow assessment and immunophenotyping [4]. In isolated extramedullary presentations without overt systemic hematologic abnormalities, tissue biopsy may be considered; however, in our patient, the hematologic findings alone sufficed without the need for invasive procedures [5]. Collaboration among hematologists, ENT specialists, and pediatricians is essential to ensure timely diagnosis and avoid unnecessary delays in initiating systemic therapy.

Extramedullary leukemic infiltrates often regress rapidly following induction chemotherapy targeting the underlying AML, as observed both in gingival hyperplasia associated with AML-M5 and rare soft tissue infiltrates [2,5]. The prognostic impact of extramedullary disease at presentation remains controversial; some studies suggest that extramedullary involvement may not independently predict poorer overall survival with modern therapy, whereas others associate it with high white cell counts and certain cytogenetic abnormalities [6,7]. Additionally, specific molecular features have been implicated in patterns of tissue infiltration and may carry separate prognostic implications [8].

This case highlights the importance of clinical vigilance. Tonsillar and other lymphoid hypertrophies with atypical features - such as rapid growth, absence of infection, and associated systemic complaints - should prompt hematologic evaluation even in the absence of classic leukemia symptoms. Early diagnosis and timely initiation of systemic therapy are critical to optimizing outcomes, minimizing complications related to airway obstruction, and ensuring comprehensive patient care [9,10].

## Conclusions

This case highlights a rare presentation of AML-M5 in a child with trisomy 21, manifesting as severe OSAS due to bilateral tonsillar infiltration. It underscores the importance of heightened clinical vigilance when assessing atypical or rapidly progressive tonsillar hypertrophy, particularly in patients with underlying predisposing conditions or unusual clinical features, as early hematologic evaluation can prevent diagnostic delays and improve outcomes. Furthermore, the rapid regression of extramedullary disease following systemic chemotherapy emphasizes the critical role of timely, multidisciplinary management in optimizing prognosis and overall patient care.
